# Examining the Effects of Chromatic Aberration, Object Distance, and Eye Shape on Image-Formation in the Mirror-Based Eyes of the Bay Scallop *Argopecten irradians*

**DOI:** 10.1093/icb/icw099

**Published:** 2016-08-22

**Authors:** Daniel I. Speiser, Yakir Luc Gagnon, Raghav K. Chhetri, Amy L. Oldenburg, Sönke Johnsen

**Affiliations:** 1 *Department of Biological Sciences, University of South Carolina, Columbia, SC, USA; † Queensland Brain Institute, Queensland University, Brisbane, Australia; ‡ Department of Physics & Astronomy, University of North Carolina, Chapel Hill, NC, USA; § Department of Biology, Duke University, Durham, NC, USA

## Abstract

The eyes of scallops form images using a concave spherical mirror and contain two separate retinas, one layered on top of the other. Behavioral and electrophysiological studies indicate that the images formed by these eyes have angular resolutions of about 2°. Based on previous ray-tracing models, it has been thought that the more distal of the two retinas lies near the focal point of the mirror and that the proximal retina, positioned closer to the mirror at the back of the eye, receives light that is out-of-focus. Here, we propose three mechanisms through which both retinas may receive focused light: (1) chromatic aberration produced by the lens may cause the focal points for longer and shorter wavelengths to fall near the distal and proximal retinas, respectively; (2) focused light from near and far objects may fall on the distal and proximal retinas, respectively; and (3) the eyes of scallops may be dynamic structures that change shape to determine which retina receives focused light. To test our hypotheses, we used optical coherence tomography (OCT), a method of near-infrared optical depth-ranging, to acquire virtual cross-sections of live, intact eyes from the bay scallop *Argopecten irradians*. Next, we used a custom-built ray-tracing model to estimate the qualities of the images that fall on an eye’s distal and proximal retinas as functions of the wavelengths of light entering the eye (400–700 nm), object distances (0.01–1 m), and the overall shape of the eye. When we assume 550 nm wavelength light and object distances greater than 0.01 m, our model predicts that the angular resolutions of the distal and proximal retinas are 2° and 7°, respectively. Our model also predicts that neither chromatic aberration nor differences in object distance lead to focused light falling on the distal and proximal retinas simultaneously. However, if scallops can manipulate the shapes of their eyes, perhaps through muscle contractions, we speculate that they may be able to influence the qualities of the images that fall on their proximal retinas and—to a lesser extent—those that fall on their distal retinas as well.

## Introduction

**Fig. 1:**
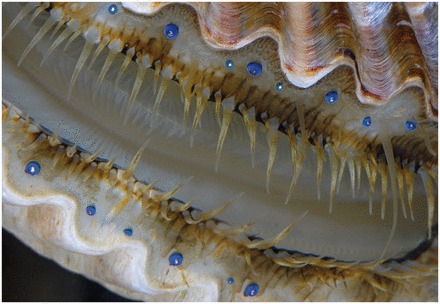
The bay scallop *Argopecten irradians*. Note the numerous eyes arrayed along the mantle margins of both valves.

Appearing along the edges of the valves by the dozens, the eyes of scallops (Family Pectinidae; Waller 2006) are a surprising sight to those not expecting to find complex visual organs in a bivalve (Fig. 1). These eyes have also held a number of surprises for researchers interested in the structure and function of visual systems: they are among the only eyes that use a concave spherical mirror to focus light for image-formation; they are one of the very few eyes to contain two separate retinas; and they provide scallops with visual acuity that far exceeds that which is observed in other bivalves. In the following article, we present a new ray-tracing analysis of the optics of these unique eyes and ask if there are scenarios in which chromatic aberration or differences in object distance may cause focused light to fall on both retinas simultaneously. We also explore whether scallops may determine which of their retinas receives focused light by altering the shapes of their eyes.

The eyes of scallops are positioned at the tips of short, flexible stalks and it has been known for over a century that each eye contains a cornea, a biconvex lens, two separate retinas, and a concave mirror (Patten 1886; Hesse 1901; Dakin 1910). Prior to work by Land (1965), it was assumed that the eyes of scallops, like the single-chambered eyes of other aquatic animals, formed images using camera-type optics in which the lens provides most of the focusing power. Working with *Pecten maximus*, Land (1965) demonstrated that the lenses of scallops lack the refractive power to focus light on to either of the two retinas and that it is the mirror at the back of the eye that is responsible for image-formation. Scallops were the first animals shown to use a concave spherical mirror for image-formation, a list that has since expanded to include the spookfish *Dolichopteryx longipes* (Wagner et al. 2009) and certain podocopid ostracods (Andersson and Nilsson 1981).

Scallops are not the only animals to have eyes with multilayer retinas—others include the firefly squid *Watasenia scintillans* (Michinomae et al. 1994), certain jumping spiders (Land 1969), and certain deep-sea fish (Denton and Locket 1989)—but they are among the few animals in which layered retinas within the same eye appear to function independently. Synaptic connections have not been identified between the photoreceptors of the distal and proximal retinas, either within the eyes (Barber et al. 1967) or within the ganglion to which the axons from both sets of photoreceptors project (Wilkens and Ache 1977; Spagnolia and Wilkens 1983). It is also likely that the two retinas gather different types of information about light: the distal photoreceptors depolarize in response to sudden decreases in light, whereas the proximal photoreceptors depolarize to a degree proportional to light intensity (Hartline 1938; Barber et al. 1967; Wald and Seldin 1968; McReynolds and Gorman 1970). Further, in *P. maximus*, the distal retina responds to moving objects, but not stationary ones, and does not provide information about the absolute intensity of light; in contrast, the proximal retina is not motion-sensitive, but does provide information about light intensity (Land 1966a).

For a non-cephalopod mollusk, scallops have eyes that provide fine spatial resolution (Table 1). Through electrophysiological experiments, Land (1966a) found that the eyes of the scallop *P. maximus* respond to dark moving stripes with angular widths as narrow as 2°, a finding consistent with earlier behavioral estimates of visual acuity in the scallop *Pecten jacobaeus* (Buddenbrock and Moller-Racke 1953). It is thought that these electrophysiological and behavioral responses are associated with spatial information collected by the distal retina: ray-tracing models by Land (1965) indicate that the distal retina lies near the focal point of the mirror and that proximal retina, positioned closer to the mirror, receives light that is out-of-focus.

**Table 1:** The visual acuities of selected mollusks, as expressed by inter-receptor angles (given in degrees). In the column titled “Method,” A and B indicate that visual acuity was estimated through anatomical or behavioral studies, respectively

Common name	Species	Inter-receptor angle (deg.)	Method	References
Octopus	*Octopus vulgaris*	0.02	A	Young (1962)
Octopus	*Octopus* sp.	0.07	B	Muntz and Gwyther (1988)
Squid	*Japetella* sp.	0.25	A	Sweeney et al. (2007)
Conch	*Strombus raninus*	0.5	A	Seyer (1994)
Scallop	*Argopecten irradians*	2	A, B	Speiser and Johnsen (2008a, 2008b)
Winkle	*Littorina littorea*	2	A	Seyer (1992)
Thorny oyster	*Spondylus americanus*	4	A	Speiser and Johnsen (2008a)
Nautilus	*Nautilus pompilius*	6.5	A, B	Muntz and Raj (1984)
Chiton	*Acanthopleura granulata*	10	A, B	Speiser et al. (2011a)
Giant clam	*Tridacna maxima*	17	A,B	Land (2003)
Slug	*Arion rufus*	26	B	Zieger et al. (2009)
Ark clam	*Barbatia cancellaria*	30	A	Nilsson (1994)

If the distal retinas of scallops are responsible for spatial vision, what is the function of the proximal retinas? Indirect evidence suggests that they play a significant role in the scallop visual system. First, the proximal retinas may account for up to 1 million photoreceptors per animal: individuals have dozens to hundreds of eyes and each proximal retina contains ∼10,000 photoreceptors (Dakin 1910, reporting on *P. maximus*). Second, the photoreceptors of the proximal retinas of scallops tend to be more tightly packed than those of the distal retinas (Speiser and Johnsen 2008a). Third, activity in the optic lobes of scallops is associated predominantly with responses to light by the proximal photoreceptors (Wilkens and Ache 1977).

The proximal retina’s relatively densely-packed photoreceptors and its association with visual processing can be justified if it receives well-focused light. To test this possibility, we evaluated three mechanisms through which both retinas in the scallop eye may receive focused light: (1) chromatic aberration produced by the lens may cause the focal points for longer and shorter wavelengths to fall near the distal and proximal retinas, respectively; (2) focused light from near and far objects may fall on the distal and proximal retinas, respectively; and (3) the eyes of scallops may be dynamic structures that determine which retina receives focused light by changing shape, a possibility supported by the eye-stalks of scallops containing longitudinal muscle fibers whose contractions are associated with the eyes withdrawing or bending away from touch or bright light (Patten 1886; Dakin 1910: Barber et al. 1967).

To test our three hypotheses, we examined eye morphology in the bay scallop *Argopecten irradians* using optical coherence tomography (OCT), a method of broadband, near-infrared interferometry that allows non-invasive, virtual cross-sectioning of live, intact biological samples (Huang et al. 1991). Within these samples, OCT reveals inhomogeneities in refractive index that exist within or between separate structures. Hence, OCT has been widely developed for human ophthalmology (Izatt et al. 1994; Hee et al. 1998). Next, we developed a computer model that traces rays of light as they interact with all of the optically significant structures of the scallop eye (i.e., the cornea, lens, retinas, and mirror). We then combined new morphological data from OCT with our computer model to predict the qualities of the images that fall on the distal and proximal retinas as a function of the wavelengths of light entering the eye (400–700 nm), object distances (0.01–1 m), and the overall shape of the eye itself.

## Methods

### Light microscopy

We collected specimens of the bay scallop *A. irradians* from either Beaufort, NC, USA (34.72°N, 76.66°W) or Smyrna, NC, USA (34.76°N, 76.53°W). Prior to dissection, we anesthetized specimens for several hours in a 1:1 aqueous solution of 3.2% NaCl and 7.5% MgCl_2_. We excised relatively large eyes (i.e., those with transverse diameters ∼0.7–1.0 mm) from the ventral sides of animals, fixed them for durations between 0.5 and 48 h, and stored them in either 70% EtOH or phosphate buffered saline with 0.01 g of sodium azide added per 50 ml as a preservative. We varied fixation time and storage conditions to test if these factors influenced the appearance of sectioned samples. We sectioned fixed eyes using a cryostat microtome (Leica Reichert-Jung Cryocut 1800) and imaged them using a Zeiss Lumar V12 stereoscope operated via a Zeiss 29D Aria workstation and AxioVision 4.6.1.0 software. In total, we gathered data for 20 eyes from three separate individuals. Following methods described previously by Speiser and Johnsen (2008a), we also fixed and sectioned 16 eyes from five separate individuals and imaged them using confocal microscopy.

### Optical coherence tomography (OCT)

The eyes of scallops are soft and prone to deformation even after they have been fixed. To avoid potential artifacts caused by fixation and sectioning, we imaged living eyes from scallops using OCT. For OCT, bay scallops (*A. irradians*) were either supplied by Gulf Specimen Marine Lab in Panacea, FL, USA (30.02°N, 84.39°W) or collected from Smyrna, NC, USA (34.76°N, 76.53°W). We kept these specimens at Duke University in a 950-liter flow-through seawater system maintained at a temperature of 20 °C and a salinity of 32 ppt (Instant Ocean sea salt, Aquarium Systems Inc., Mentor, OH, USA). We then transported specimens by car to the University of North Carolina at Chapel Hill. There, we dissected a strip of mantle tissue several millimeters long from the ventral region of the left valve of each specimen and affixed it, with insect pins, to the wax-coated bottom of a small, seawater-filled dish. We positioned the mantle tissue so that the eyes faced upward. To minimize optical dispersion from the water, we adjusted the water level in the dish with a dropper to just cover the eyes. We observed that these pieces of tissue remained alive—that is, responsive to touch and bright light—for several hours following excision.

We performed OCT using a spectral-domain system described previously (Oldenburg et al. 2010), with minor modifications. Specifically, we used a light source centered at a wavelength of 800 nm with a bandwidth of ∼120–130 nm and an imaging lens with a focal length of 30 mm that provided a resolution of ∼12 × 3 µm (lateral × axial) in water. In all cases, we adjusted the position of our tissue sample so that we captured a virtual cross-section through the center of an eye, which we defined as the position at which the diameter of the pupil was maximal. Values for axial line-rates, power in the sample, and exposure time are provided in Table 2. We also generated 3D images of eyes from certain specimens (see Table 2). Here, we used steps in *y* of 50 or 5 µm and collected 11 *x-z* images (10 steps total) or 51 images (50 steps total), respectively, giving 3D images with spatial extents (*x *×* y* × *z*) of 1 × 0.5 × 1.5 mm (specimen 1) or 1.72 × 0.25 × 1.5 mm. We performed OCT at 21 °C under dim room lights, conditions neither unnaturally warm nor bright for the shallow-dwelling *A. irradians.*

**Table 2:** Information describing scallop eye OCT

Specimen	No. of eyes examined	Axial line rates (kHz)	Power in the sample (mW)	Exposure time (µs)	Sample dimensions (*x* x *z*) in mm	Sample dimensions (*x* x *z*) in no. of pixels	Collection, Location
1*	5	5	7	190	1.00 x 1.56	1000 x 1024	Panacea, FL
2	5	5	14	30	1.72 x 1.56	1000 x 1024	Panacea, FL
3*	4	5	14	30	1.72 x 1.56	1000 x 1024	Panacea, FL
4	13	25	7	38	1.56 x 1.56	1024 x 1024	Smyrna, NC
5	21	25	6.5	38	1.56 x 1.56	1024 x 1024	Smyrna, NC

*Denotes samples for which 3D images were gathered.

To calculate true distances from distances measured along the *z*-axes of our OCT images, we accounted for optical path delay through materials with different refractive indices. OCT measures optical path length, which is the physical path length times the refractive index of the material being imaged. We calibrated physical path length in free space using a micrometer to translate an object and track the number of pixels that it moved, resulting in a calibration of physical distance (in µm) equaling the number of pixels × 2.021 µm. For structures within scallop eyes with the same refractive index as seawater at 800 nm (*n* = 1.334), we calculated that the physical distance (in µm) equals the number of pixels × 1.515 µm. For structures with a different refractive index than seawater at 800 nm, we calculated that physical distance (in µm) equals the number of pixels × 2.021 µm, divided by the refractive index of the structure. To measure the refractive indices of isolated scallop corneas, lenses, and retinas over the wavelength band of the OCT system, we followed a method described previously (Tearney et al. 1995). Briefly, the method entails dissecting a small (∼100–500 µm) layer of tissue of interest, placing it on a planar surface such as a microscope slide, and observing the apparent deviation of the planar surface within the OCT image. This deviation is attributed to the optical path delay induced by the intervening sample, from which the effective refractive index can be calculated. Through this procedure, we found that the retinas and corneas of *A. irradians* appeared to have refractive indices similar to that of seawater (within experimental error) at the wavelengths of the OCT system, but that the lens had a refractive index of ∼1.35 at these wavelengths.

We interpreted our results from OCT with the understanding that this imaging method only detects singly-backscattered light that is still coherent with the incident light; multiply-scattered light loses this coherence and is filtered out, enabling the imaging of several mean free scattering path lengths into tissue. Thus, we expected to see back-scatter from boundaries between layers with different refractive indices (*n*) or within layers that are turbid*—*that is, have inhomogeneities in refractive index within and between cells of the same type. Within the eyes of scallops, we predicted such scattering to occur at the interface between the seawater medium and the cornea, between the cornea and the lens, and between the lens and retina. We also expected to see back-scatter from the mirror at the rear of the scallop eye, as well as sub-cellular structures such as nuclei and mitochondria.

### An optical model for the scallop eye

To describe the optical performances of scallop retinas as functions of the wavelengths of light entering an eye, object distances, and the general shape of the eye, we used a novel ray-tracing method that is especially well-suited for analyzing the performance of animal eyes because it allows multiple atypical surfaces to be described and traced easily. This method—developed by Gagnon et al (2014) —represents rays of light and optical interfaces as a continuous function by utilizing Chebyshev approximations (via the chebfun toolbox in Matlab, R2011b, Mathworks Inc., Natick, MA, USA). We modeled the wavelength dependence of the refractive index of ocular media using a previously published model that was based on direct measurements from vertebrate eyes and then generalized for broader application to other optical systems (Gagnon et al. 2010). We examined wavelengths from 400 to 700 nm because neither the distal nor the proximal photoreceptors of *A. irradians* appear to have significant sensitivity to UV or IR wavelengths (McReynolds and Gorman 1970; Speiser et al. 2011b). We modeled object distances between 0.01 and 1 m (relative to the surface of the cornea) under the assumption that objects at closer distances would likely be in direct physical contact with the scallop and that longer object distances would not affect image quality significantly given the relatively coarse spatial resolution of scallop eyes (Table 1). Lastly, we modeled changes in the shape of scallop eyes by setting the volume of eyes constant and then calculating their width as a function of their length. Our transformations involved multiplying the *y*-axis of an eye by a number that we will refer to as a morph factor and then rescaling the *x*-axis of the eye by the reciprocal of the square root of this morph factor (i.e., the volume of the eye was set to equal that of a cylinder). Thus, an eye with a morph factor of 1 has dimensions that correspond to our empirical measurements (Table 3) and eyes with morph factors greater than or less than 1 are elongated along their axial or transverse axes, respectively.

**Table 3:** The values we used as inputs for our ray-tracing model of image-formation in the eyes of the bay scallop *A. irradians*

Model parameter	Value(s)	Source
Wavelength (nm)	400–700	N/A
Object distance (m)	0.01–1	N/A
Morph factor	0.73–1.17	N/A
Aperture (µm)	251	OCT
Photoreceptor width (µm)	5	Speiser and Johnsen (2008a)
Axial lengths (µm)		
Distance between the distal and proximal surfaces of the cornea	23	Light microscopy
Distance between the distal surface of the cornea and the proximal surface of the lens	238	OCT
Distance between the distal and proximal surfaces of the lens	215	Light microscopy/OCT
Distance between the proximal surface of the lens and the mirror	136	OCT
Lengths of the cilliary projections from the distal photoreceptors	12	Speiser and Johnsen (2008a)
Lengths of the rhabdoms of the proximal photoreceptors	30	Speiser and Johnsen (2008a)
Total axial length of the distal and proximal retinas	102	Light microscopy
Distance between the distal surface of the cornea and the mirror	374	OCT
Refractive indices (*n*)		
Cornea	1.37	Sivak and Mandelman (1982) for human cornea
Lens	1.42	Land (1965) for the lens of the scallop *Pecten maximus*
Distal retina	1.35	Sivak and Mandelman (1982) for cytoplasm
Proximal retina	1.35	Sivak and Mandelman (1982) for cytoplasm
Gap	1.34	Sivak and Mandelman (1982) for human vitreous humor
Radii of surface curvatures (µm)		
Cornea	See text	Light microscopy
Distal lens	See text	Light microscopy
Proximal lens	337	Light microscopy
Distal retina	337	Light microscopy
Proximal retina	337	Light microscopy
Mirror	417	Light microscopy

*Note*: As described in the text, “Morph factor” refers to how we modeled scallop eyes with equal volumes, but different shapes. Eyes with morph factors >1 are elongated along their axial axis and those with morph factors <1 are elongated along their transverse axis.

We predicted the qualities of the images received by the distal and proximal retinas of scallops by calculating Point Spread Functions (PSFs). The PSF of an eye describes the image formed on its retina by a distant point source. In an ideal optical system, acuity is limited only by diffraction: a point source forms an Airy disk whose width is determined by the ratio of the diameter of the eye’s pupil to the wavelength of the light entering it (Land and Nilsson 2002). In the case of the scallop eye, diffraction amounts to a PSF that is ∼0.13° wide at half its height (i.e., full width at half maximum, or FWHM, a measurement we will explain in greater detail below). However, the eyes of animals are not ideal optical systems and the PSFs of the images they form depend on many other factors that increase the size of the PSF and thus reduce the quality of the image. These factors include the curvatures of the surfaces within an eye, the distributions of refractive indices within or between these surfaces, and the degree to which any of these refractive indices are wavelength-dependent. Despite these computational challenges, the PSFs of many biological optical systems can be approximated as 3D Gaussian functions in which the distribution of light intensity on the retina follows a bell-shaped curve (Land and Nilsson 2002). The width of the Gaussian PSF at half its maximum (FWHM) is inversely related to an eye’s acuity, that is, the amount of spatial information that it can transfer from an object to its retina. We calculated PSFs and their corresponding FWHMs because it is a relatively straightforward way to study how different viewing conditions and morphological parameters may influence an eye’s performance.

To calculate the PSFs of the scallop’s two retinas, we set each retina's nodal point to the center of the circle that best fit the curvature of the mirror, the most important optical component of the eye. The nodal points of an optical system can be defined in a number of functionally-dependent ways (Hecht 2002). For the current study, we define a nodal point as the optical center of the system; or, more specifically, the point around which a scene or signal can rotate with minimal effect on the image formed (Hecht 2002). Using this definition, we derived the angular PSFs of the scallop’s distal and proximal retinas by dividing the widths of their respective PSFs by the distances between their physical positions and their nodal points.

### Morphological parameters for our model

The inputs for our ray-tracing model include: (1) the aperture of the pupil; (2) the refractive indices and curvatures of the surfaces of the scallop eye, which include the cornea, lens, distal retina, proximal retina, and mirror; and (3) the axial lengths of the structures within the scallop eye. We used images from OCT to estimate pupil aperture and, whenever possible, the axial lengths of the structures within the eyes (Table 3). While our OCT images of living eyes were appropriate sources for estimating certain axial lengths, we relied on images from light and confocal microscopy to obtain the axial lengths of the cornea and the photoreceptive regions of the distal and proximal photoreceptors. We were also unable to use OCT data to measure the curvatures of layers beneath the cornea because the apparent curvatures in the images are influenced by the curvatures of any overlying layers with different refractive indices. Instead, we estimated curvatures of the cornea and the distal surface of the lens by fitting the modified Lorentzian function,

$$d+\frac{c}{{\left(\frac{x^{2}}{a^{2}}\right)}^{b}+1}$$

to images of sectioned eyes that had been fixed for a minimal amount of time (0.5–4 h) and stored in phosphate-buffered saline (PBS). We preferred to work with these samples because we have observed that longer fixation times and/or storage in dehydrating media causes lenses to become misshapen. We chose a Lorentzian function because it resulted in the best fit to the imaged curves when compared to similar types of functions (e.g., Gaussian, aspherical, polynomial, etc.). For our calculations we used values for *a, b, c*, and *d* of 150.2, 0.9, 124.0, and −92.0, respectively, for scallop corneas and corresponding values of 125.4, 0.6, 120.8, and −67.1 for scallop lenses. We took a similar approach to estimating the curvatures of the proximal surface of the lens, distal and proximal retinas, and mirror, but fit hemispherical curves to these surfaces instead. Finally, for the refractive indices of the various layers of the scallop eye (at 550 nm) we assumed the following: that the cornea has a refractive index of 1.37; that the lens has a uniform refractive index of 1.42 (*P. maximus*; Land 1965); and that the retinas have a refractive index of 1.35 (Table 3).

## Results

### Live imaging of scallop eyes using OCT

**Fig. 2:**
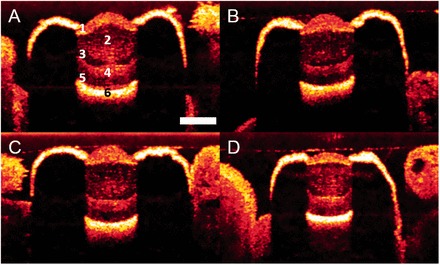
Virtual axial cross-sections of live scallop eyes acquired using OCT. Panel A displays an eye in which labels have been applied to the six scattering bands visible in the majority of our OCT images. Similar scattering bands may be seen in panels B, C, and D. We propose the following interpretation of our OCT images: band 1 represents the cornea, the thin layer of connective tissue between the cornea and lens, and the distal portion of the lens; band 2 represents the remaining area of the lens; band 3 corresponds to the ciliary projections of the distal retina; band 4 represents the cell bodies of the distal and proximal photoreceptors, as well as the glial cells that lie between them; band 5 contains the rhabdoms of the proximal retina and—potentially— a fluid-filled gap between the rhabdoms and the mirror at the back of the eye; band 6 is back-scatter from the mirror. All four panels represent separate eyes imaged under similar conditions. The scale bar in panel A represents 200 µm and applies to all four panels.

We used OCT to acquire virtual cross-sections of live, intact eyes from the bay scallop *A. irradians* (Fig. 2; also see Supplemental Video 1 for a 3D reconstruction). The OCT beam passed through the pupil and reached the mirror at the back of the eye, but did not penetrate the heavily pigmented epithelial layer that shrouds the rest of the eye. The eyes we imaged using OCT had maximum transverse diameters of 700 ± 13 µm (mean ± std error) and pupils with diameters of 250 ± 6 µm (*N = *36 for both measures).

Separate layers of tissue within the eyes of *A. irradians* scattered the OCT beam to different degrees, possibly due to differences in their sub-cellular morphologies. Here, our results are broadly similar to those from OCT investigations of human eyes, in which separate tissue layers may be distinguished by the amount of scattering they cause (Hee et al. 1995; Drexler et al. 2001). The distal-most surfaces of scallop eyes were marked by a band of heavy back-scatter (band 1; Fig. 2A) with an axial length of 53 ± 2 µm (*N *= 36). We find it likely that this first band of back-scatter represents the cornea, the thin layer of connective tissue between the cornea and lens, and the distal portion of the lens. Next, we noted a region of moderate back-scatter (band 2; Fig. 2A) consistent with the size and location of the lens. The cells that comprise the corneas and lenses of scallops contain nuclei and other organelles (Barber et al. 1967), so we suspect that the cornea was associated with more back-scatter than the lens because the cells of the former are packed more tightly than those of the latter (Speiser and Johnsen 2008a).

The distal surface of band 1 and the proximal surface of band 2 were relatively unambiguous landmarks in our OCT images (Fig. 2A). If we assume that the distal surface of band 1 marks the distal surface of the cornea and that the proximal surface of band 2 marks the proximal surface of the lens, our OCT images indicate that the axial distance from the distal surface of the cornea to the proximal surface of the lens is 240 ± 7 µm (*N *= 36). Light microscopy indicates that the corneas of *A. irradians* have an axial length of 17 ± 1 µm (*N *= 36) and that there is a thin layer of connective tissue between the cornea and lens with an axial length of 6 ± 1 µm (*N *= 36). Combining information from OCT and light microscopy, we conclude that the lenses of *A. irradians* have an axial length of ∼220 µm.

Moving deeper into the eye, we noted bands of scatter that are consistent with the separate tissue layers of the scallop retina. Immediately proximal to the lens, there was a band of low scatter (band 3; Fig. 2A) with an axial length of 37 ± 3 µm (*N *= 36) and a position that corresponds to the ciliary projections from the photoreceptors of the distal retina. These ciliary projections are ∼12 µm in length in *A. irradians* (Speiser and Johnsen 2008a) and are of similar length in other species of scallop such as *P. maximus* (Barber et al. 1967). Immediately proximal to band 3, we detected a region of moderate scatter (band 4; Fig. 2A) with an axial length of 35 ± 2 µm (*N *= 36) that corresponds to the size and position of the region of the scallop retina that contains the cell bodies of the distal and proximal photoreceptors, as well as the glial cells that lie between them (Barber et al. 1967).

Toward the back of the eye, we found a region of low scatter (band 5; Fig. 2A) followed by a band demonstrating the highest levels of back-scatter that we observed in our OCT images (band 6; Fig. 2A). We interpret the latter of these two bands (band 6) as back-scatter from the mirror at the back of the eye. The mirror in the scallop’s eye is a multi-layer reflector (or Bragg stack) that produces high reflectance due to constructive interference between reflections from all of the internal interfaces of the multiple layers that comprise it (Land 1966b). While conducting OCT, we noted that scattering by the mirror was maximal at the very center of the eye (in some cases overwhelming the signal from other structures within the eye). Conversely, when eyes were tilted with regard to the beam, we saw very little back-scatter because rays were not coming straight back to the detector through the pupil.

If we interpret the proximal surface of band 2 as the proximal surface of the lens and the distal surface of band 6 as the distal surface of the mirror, the axial distance between the distal retina and the surface of the mirror averaged 140 ± 5 µm (*N = *36). We know that this region of the eye contains the ciliary projections of the distal photoreceptors (corresponding to band 3), the cell bodies of the distal and proximal photoreceptors (band 4), and the rhabdoms of the proximal photoreceptors. These rhabdoms likely account for some of the axial length of band 5. However, we know that these rhabdoms are ∼30–40 µm in length in *A. irradians* (Speiser and Johnsen 2008a), which leaves some of the space (∼30–40 µm) between the lens and mirror unaccounted for. Thus, we expect that there is a fluid-filled gap between the proximal retina and the mirror. Given that back-scatter has been observed from the photoreceptive regions of rods and cones in OCT investigations of vertebrate retinas (Hee et al. 1995; Drexler et al. 2001), we were surprised to see little scattering from either the ciliary projections of the distal photoreceptors or the rhabdoms of the proximal photoreceptors. However, these folds might be too fine to scatter the OCT beam or the scattering observed in vertebrate retinas may come from sources not present in the photoreceptors of scallops.

### Modeling image formation within the scallop eye

**Fig. 3:**
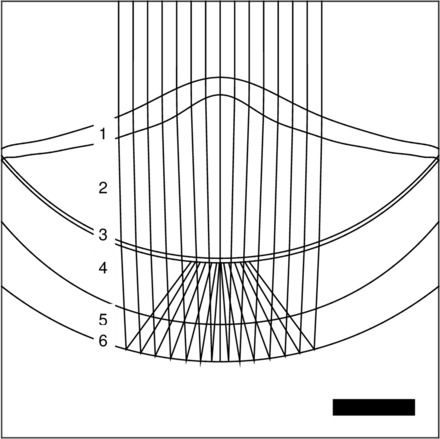
An example of the ray-tracing model we used to predict the qualities of the images received by the distal and proximal retinas of scallops given different conditions. Here, the structures within the scallop eye are labeled as follows: 1 – cornea; 2 – lens; 3 – the ciliary projections of the distal photoreceptors; 4 – the cell bodies of the distal photoreceptors, the glial cells that lie between the two retinas, and the proximal photoreceptors; 5 – an inferred gap between the proximal retina and the mirror; 6 – the concave mirror at the back of the scallop eye. The scale bar represents 100 µm.

We used a custom-built ray-tracing model to estimate the qualities of the images received by the distal and proximal retinas of *A. irradians* (Fig. 3). When we assume 550 nm wavelength light and an object distance greater than 0.01 m, our computer model indicates that a hypothetical eye from *A. irradians* (see Table 3) has an angular resolution of 2° (FWHM of 2°) at its distal retina and a lower angular resolution of 7° (FWHM of 7°) at its proximal retina. Next, we tested three mechanisms through which both the distal and proximal retinas could receive sharply-focused images. We used our computer model to ask: (1) whether longitudinal chromatic aberration (LCA) caused by the refractive components in the scallop eye could place focused light of different wavelengths on the distal and proximal retinas simultaneously; (2) if focused images of near and far objects fall on the distal and proximal retinas, respectively; or (3) if small changes in the overall shape of the eye could determine whether focused images fall on the distal or proximal retina.

**Fig. 4:**
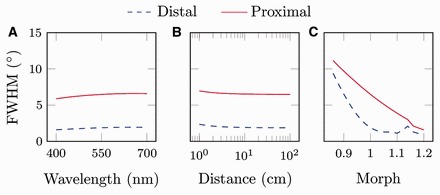
The influences of (A) wavelengths of light entering the eye, (B) object distances, and (C) morph factors on the FWHM of the distal (dashed line) and proximal (solid line) retinas of the eye of the bay scallop *A. irradians*. Here, morph factor refers to the degree to which an eye is elongated with regard to our empirical measurements. An eye with a morph factor of 1 has dimensions that correspond to our empirical measurements. Eyes with morph factors > 1 are elongated along their axial axis; eyes with morph factors < 1 are elongated along their transverse axis.

Our results do not support our first or second hypotheses, but our model indicates that small changes in the shape of an eye from *A. irradians* could influence the qualities of the images that fall on the distal and proximal retinas by moving the retinas closer to or further away from the focal point of the eye. Our computer model rejects our first hypothesis by indicating that the distal and proximal retinas have angular resolutions of 1.6° and 5.9°, respectively, when 400 nm light enters the eye and very similar angular resolutions of 1.9° and 6.6°, respectively, when 700 nm light enters the eye (Fig. 4A). In other words, due to the relatively low refractive index (and thus low dispersion) of the scallop lens, there is little separation between images composed of long- and short-wavelength light. Results from our computer model lead us to reject our second hypothesis as well. We find that the distal and proximal retinas have angular resolutions of 2.4° and 7.0°, respectively, when viewing objects at a distance of 0.01 m; similarly, the distal and proximal retinas have angular resolutions of 1.9° and 6.5°, respectively, when viewing objects at a distance of 1 m (Fig. 4B). Finally, we used our computer model to test if scallops may be able to focus light on to either their distal or proximal retinas by changing the shapes of their eyes. We find that scallops can focus light on to their proximal retinas if their eyes are able to change shape so that they are elongated slightly along their axial dimension. For example, if we model an eye that is elongated 17% in the axial dimension compared to our empirical measurements (i.e., an eye with a morph factor of 1.17), the FWHM of the proximal retina becomes ∼2° while the FWHM of the distal retina does not change appreciably (Fig. 4C).

## Discussion

### Morphology of living scallop eyes

By using OCT to image living eyes from the bay scallop *A. irradians*, we have gained new insights into two long-standing questions about scallop eye morphology: (1) the natural shape of the lens, particularly the curvature of its distal surface and (2) whether or not a gap exists between the rhabdoms of the proximal retina and the mirror at the back of the eye. With regard to the shapes of scallop lenses, we find that the lenses of different species of scallop may be more similar in shape than has been indicated by past comparative studies (e.g., Speiser and Johnsen 2008a). For example, we find that the distal surfaces of the lenses of *A. irradians* appear to be curved similarly to those of *P. maximus* (Land 1965) and *Placopecten magellanicus* (Speiser and Johnsen 2008a). Past studies of scallop eye morphology have relied on fixed, sectioned samples and we suspect that fixation may influence the shapes of scallop lenses in a species-dependent manner. Obtaining higher-resolution images of living eyes from *A. irradians* (and other scallop species) may provide further support for the hypothesis that the lenses of scallops are shaped in a way that helps to correct for the spherical aberration produced by the mirror (Land 1965).

We also find indirect evidence that a fluid-filled space may separate the rhabdoms of the proximal retina from the mirror at the back of the eye of *A. irradians.* Certain past authors have argued that this gap is a real feature of the eyes of at least certain species of scallops (Speiser and Johnsen 2008a; Salvini-Plawen 2008), whereas others have argued that any gap observed between the proximal retina and mirror is a histological artifact (Dakin 1910; Land 1965). We suspect that this gap is a real feature of the eyes of *A. irradians*. For this gap to not be implied by our OCT images, the rhabdoms of the proximal retina would have to be twice as long (∼60–80 µm) as estimated previously (∼30–40 µm; Speiser and Johnsen 2008a). Dakin (1910) reported that the rhabdoms of the proximal retina of *P. maximus* are surrounded by a matrix composed of a “semi-fluid substance of connective-tissue like nature.” We suspect that this matrix forms a layer between the proximal retina and mirror in *A. irradians*.

If a fluid-filled space lies between the proximal retina and mirror in the eyes of *A. irradians* (and perhaps in the eyes of other species as well), it may be absent in certain histological preparations because lightly-fixed scallop eyes tend to collapse during storage and/or sectioning. In a series of experiments, we found that cryo-sections of eyes from *A. irradians* that had been fixed for 0.5–4 h had proximal retinas and mirrors that were in direct contact (in 10 out of 10 samples). Conversely, cryo-sections of eyes that had been fixed for 12–48 h had gaps between the proximal retina and mirror (in 21 out of 21 samples). The presence of such a gap did not depend on whether fixed eyes had been stored in 70% EtOH (16 samples) or PBS (5 samples) after fixation. These results are consistent with our observations that living scallop eyes have relatively full shapes that tend to crumple when they are fixed for short periods of time and then dehydrated. We also observed that the shapes of the lenses of *A. irradians* are influenced by preparation methods, but that their axial lengths are not. Lenses fixed for 0.5–4 h (*N = *10) had an average axial thickness of 240 ± 12 µm; in comparison, lenses fixed for 12–48 h (*N = *21) had an average axial thickness of 250 ± 10 µm (*P* = 0.4; two-tailed *t*-test).

### Separate retinas, separate functions?

Behavioral experiments suggest that the eyes of scallops provide spatial vision with an angular resolution of ∼2º (Buddenbrock and Moller-Racke 1953; Speiser and Johnsen 2008b). Results from electrophysiology (Land 1966a) and optical modeling (Land 1965; Speiser and Johnsen 2008a) suggest that it is the distal retinas of scallops that provide the relatively fine-grained spatial vision demonstrated by animals in behavioral experiments. Here, our computer model supports these previous studies by indicating that the distal retinas of *A. irradians* receive focused light with a FWHM of ∼2º.

The role of the proximal retinas in the scallop visual system is less well-understood. It has been argued that the proximal retina lies too close to the mirror to receive focused light by reflection (Land 1965). Instead of each proximal retina perceiving an image, it has been proposed that the proximal retinas of each eye act as the individual sampling units of a dispersed compound eye that includes all of the proximal retinas of the dozens of other eyes on the mantle (Land 1968; Wilkens 2006). Our results do not rule out this possibility, but we find evidence that the proximal retinas may receive partly-focused light: the mid-points (in the axial dimension) of the rhabdoms of the proximal retinas receive light with a FWHM of 7°. Thus, our findings suggest that the proximal retinas of scallops may each contain a multitude of photoreceptors—up to 10,000 apiece—because each retina gathers at least a limited degree of spatial information.

Following past authors (Land 1966a; Speiser and Johnsen 2008a), we hypothesize that the two separate retinas in the eyes of scallops gather information relevant to specific tasks. Morphological and physiological studies indicate that synaptic connections between the distal and proximal photoreceptors are absent within the eyes of scallops (Dakin 1910; Muller 1958; Barber et al. 1967), as well as within the optic nerves that exit the eyes (Land 1966a) and the nerve center (the parietal-visceral ganglion or PVG) to which nearly all of the optic nerves project (Wilkens and Ache 1977; Spagnolia and Wilkens 1983). We propose that the hyperpolarizing receptors of the motion-sensitive distal retina may be used for the detection of moving objects, such as predators. Scallops distinguish predators from other animals using chemosensory and tactile cues (Wilkens 1981), so visual cues gathered by the distal retina may simply alert scallops to the presence of potential threats. Next, we propose that the depolarizing receptors of the motion-insensitive proximal retina may be used for habitat selection. Scallops use visual cues for habitat selection (Hamilton and Koch 1996), which implies that these animals are gathering information about static features in their environment—a task for which the tonic receptors of the proximal retina may be better-suited than the phasic receptors of the distal retina.

### Two images for two retinas?

We evaluated three mechanisms through which both retinas in the eye of *A. irradians* may receive focused light: (1) LCA produced by the lens may cause the focal points for longer and shorter wavelengths to fall near the distal and proximal retinas, respectively; (2) focused light from near and far objects may fall on the distal and proximal retinas, respectively; and (3) the eyes of scallops may be dynamic structures that determine which retina receives focused light by changing shape. Our computer model rejects our first hypothesis (proposed in Speiser et al. 2011), by indicating that it is unlikely that the scallop lens produces sufficient LCA for focused images of different wavelengths to fall on both retinas simultaneously (Fig. 4A). For LCA to have a significant influence on where focused light falls within the eyes of *A. irradians*, the lenses of this scallop would have to produce an amount of dispersion unprecedented for a biological material. By approximating the dispersion of the scallop lens using Cornu's formula (Le Grand 1956), we found that the amount of dispersion required for two different wavelengths of light (400 and 700 nm) to come to a focus on both retinas simultaneously is outside the realm of biological possibility, that is, the scallop lens would need to have an Abbe number of about 10, even lower than that of a diamond.

Dakin (1910) was the first to hypothesize that images of near and far objects may fall on the distal and proximal retinas of scallops, respectively, writing “[n]ow it may be that the two layers of recipient cells [the distal and proximal retinas] are for the reception of images situated at different distances from the eyes, which are focused at different distances from the lens.” By altering the viewing distances used as input for our computer model, we found that viewing distances of 0.01–1 m had little influence on where focused light falls in the eye of *A. irradians* (Fig. 4B)*.* We conclude that a scallop-like eye could employ two separate retinas to detect objects at different viewing distances, but that the eyes of scallops do not appear to function in this manner.

If scallops are able to voluntarily alter the shapes of their eyes, our computer model suggests that they may be able to control the qualities of the images that fall on their distal and proximal retinas (Fig. 4C). Our computer model indicates that the FWHMs associated with both retinas will decrease if an eye is elongated in the axial dimension. This is because both retinas in the scallop eye are located proximal to the true focal point of the imaging system and will move closer to the focal point as the eye elongates. Also, as the eye elongates in the axial dimension, the FWHM registered at the proximal retina will decrease more rapidly than the FWHM at the distal retina because the proximal retina is located further away from the focal point of the eye. Therefore, any elongation of the eye will have a greater effect on the quality of the image formed on the proximal retina than the quality of the image formed on the distal retina.

Our hypothesis that the eyes of scallops are dynamic structures is not unprecedented. Poli (1795) considered the eye-stalks of scallops to be modified versions of the extensible, mobile sensory tentacles with which they are interspersed on the mantle. Subsequent researchers found that the eye-stalks contain small longitudinal muscle fibers, but lack the helical fibers present in the sensory tentacles (Dakin 1910; Barber et al. 1967). Contractions of these muscle fibers in the eye-stalks are probably responsible for the eyes being able to withdraw or bend away from touch and bright light (Patten 1886; Dakin 1910: Barber et al. 1967).

As another historical example, Patten (1886) suggested that the eyes of scallops may be capable of accommodation through muscular contractions that adjust the axial distance between the lens and the retinas. Dakin (1910) rejected Patten’s hypothesis by arguing that it would not work for a camera-type eye (as the scallop eye was thought to be at the time) because the lens and retinas would remain the same distance away from each other when the eye changed shape. However, we now know that the eyes of scallops focus light by reflection (Land 1965). Thus, contractions and relaxations of the longitudinal muscle fibers ought to cause the lens and retinas to move further from or closer to the mirror, respectively, thereby controlling—at least potentially—which of the two retinas lies closer to the focal point of the eye.

In the absence of helical muscle fibers, how do the eyes of scallops return to their original shape once the longitudinal muscle fibers relax? We propose two potential sources of this elasticity. First, following Patten (1886), we hypothesize that the elasticity of the septum that separates the lens from the retinas may cause the eyes of scallops to elongate along their transverse axis when the longitudinal muscles relax. Second, the eyes of scallops may change shape hydraulically: Dakin (1910) reported that “the blood plays an important part in the extension of the tentacles, and if a small living *Pecten* is watched under the microscope, the corpuscles can be traced running rapidly along the cavities of the tentacles as they are extended and back in the reverse direction as they contract.” These two mechanisms are not mutually exclusive and both are worth further investigation.

## Supplementary Material

Supplementary Data

Supplementary Data
