# Supplementary material for: Examining the Effects of Chromatic Aberration, Object Distance, and Eye Shape on Image-Formation in the Mirror-Based Eyes of the Bay Scallop Argopecten irradians
Source: Integr Comp Biol. 2016 Aug 22;56(5):796–808. doi: 10.1093/icb/icw099 (PMC5886045; doi:10.1093/icb/icw099)
Supplement: Supplementary Data [file supp_56_5_796__index.html]

Examining the Effects of Chromatic Aberration, Object Distance, and Eye Shape on Image-Formation in the Mirror-Based Eyes of the Bay Scallop Argopecten irradians — Supplementary Data 

# Examining the Effects of Chromatic Aberration, Object Distance, and Eye Shape on Image-Formation in the Mirror-Based Eyes of the Bay Scallop *Argopecten irradians*

## Supplementary Data

files

- Supplementary Data - zip file
